# Monitoring the efficacy of tumor necrosis factor alpha antagonists in the treatment of Ankylosing spondylarthritis: a pilot study based on MR relaxometry technique

**DOI:** 10.1186/s12880-021-00646-9

**Published:** 2021-07-30

**Authors:** Mingui Lin, Xianyuan Chen, Shun Yu, Fei Gao, Mingping Ma

**Affiliations:** 1 The Shengli Clinical Medical College, Fujian Medical University, Radiology Department of Fujian Provincial Hospital, 134 East Street, Gulou District, Fuzhou City, 350001 Fujian Province China; 2 Rheumatism Department of Fujian Provincial Hospital, Fuzhou, 350001 China

**Keywords:** Tumor necrosis factor alpha, Ankylosing spondylarthritis, T1 mapping, T2 mapping, T2* mapping

## Abstract

**Background:**

SpA is a disease that seriously affects the quality of life and working ability of patients. At present, there is a lack of scientific and effective quantitative indicators to evaluate the activity of sacroilitis and the efficacy of tumor necrosis factor-α antagonists in the treatment of active sacroilitis. MRI STIR sequence is the most commonly used method for the diagnosis of sacroiliac joint inflammation, but its response to the disease still lags behind the pathological changes and cannot provide quantitative indicators. This study aimed to evaluate the feasibility of using MRI Relaxometry technique to monitor the efficacy of TNF-α antagonists in the treatment of SpA, so as to provide an effective quantitative index for monitoring the efficacy.

**Methods:**

This is a prospective study, 114 patients with sacroiliac joint were enrolled, including 15 patients as a control group, 99 patients as the case group, and 20 patients in the case group as the treatment group. The differences of T1 mapping, T2 mapping, T2* mapping of subchondral bone marrow of sacroiliac joint were compared among different groups. The diagnostic efficacy was analyzed by ROC, and the best quantitative index of diagnostic efficiency was used to monitor curative effects of different treatment cycles in the treatment group.

**Results:**

1. Compared with the control group, values of three different relaxation times in the subchondral bone marrow region of the sacroiliac joint in the case group increased in varying degrees, and T1 mapping showed the best diagnostic efficacy. 2. The decreasing rate of T1 mapping in different treatment periods benefits the monitoring of curative effects.

**Conclusion:**

This study indicates that T1 mapping technique is preferred in quantitative diagnosis. T1 mapping is superior to T2* mapping and T2 mapping in the diagnosis of subchondral BME of SpA. It can quantitatively monitor edema changes during treatment, benefiting clinical individualized treatment and timely adjustment of the treatment plan.

## Introduction

SpA (Ankylosing spondylarthritis), belonging to serum-negative spondyloarthritis, is a chronic inflammatory disease which typically affects the sacroiliac joints and the spine. Early sacroiliac joint involvement is the characteristic of SpA [1]. The presence of active inflammation is both a key component of the diagnosis of SpA and a target for therapy. BME (Bone marrow edema) of the sacroiliac joint is considered the main imaging marker of this inflammation. Such disease is characterized by younger age of onset, high prevalence rate, long course of disease and recurrent disease. In severe cases, it can cause ankylosis of sacroiliac joint and axial spine, which greatly affects living quality and working ability of patients. TNF-α antagonists can effectively control inflammation, reduce the active state of SpA disease, delay the progression of SpA, and improve the prognosis [2]. However, there is a lack of scientific and effective quantitative indicators to evaluate the efficacy of TNF-α antagonists in the treatment of active sacroiliitis. Therefore, it is significant to find a method for quantitative evaluation of sacroiliac joint inflammation and curative effect evaluation. Quantified MRI parameters [3–5], including T1 mapping, T2 mapping, and T2* mapping, can reflect changes of water content in bone marrow by detecting small changes of water molecules in tissue, and quantify the degree of subchondral BME of sacroiliac joint, which is beneficial to early diagnosis and monitoring the condition of bone marrow lesions. In this study, MR Relaxometry technique was used to quantitatively evaluate the changes of subchondral BME of sacroiliac joint in SpA to provide scientific and effective quantitative indexes for clinical diagnosis, activity staging, curative effect evaluation and monitoring of SpA.

## Materials and methods

### General information

From October 2017 to May 2020, 114 patients participated in this study. The inclusion criteria of the case group were: (1) patients were enrolled following the classification standard of axial spondyloarthropathy issued by ASAS (Assessment of Spondyloarthritis International Society) in 2009 [6]; (2) patients were diagnosed as AS according to the revised New York standard [7], with low back pain lasting longer than one year; and (3) clinical and MRI data of patients were complete. The inclusion criteria of the control group were as follows: (1) sacroiliac joint MRI examination was performed in patients with simple chronic low back pain; and (2) there was no high signal intensity in the bilateral sacroiliac joint bone in STIR sequence, and SpA diagnosis was excluded. All subjects with MRI contraindications were excluded. Subjects were also excluded for having primary osteoporosis, metabolic system disease, immune system disease, tumor or cancer, combination with other bone diseases, and hormone and immune drug medication in the past 6 months.

### Experimental grouping

The study involved 114 patients (69 Male and 45 Female; mean age, 35.3 years; range, 14–71 years), including 15 normal sacroiliac joint patients without SpA as a control group, 99 patients with clinically diagnosed SpA as the case group. 20 patients in the case group treated with systematic TNF-α antagonists (Enbrel, once a week, 50 mg) formed the treatment group. According to the score of ASDAS-CRP, the case group was divided into an Active group and an Inactive group. The Activity group was divided into 3 subgroups: moderate activity group (1.3–2.1), high disease activity group (2.1–3.5), and very high disease activity group (> 3.5). According to different times of treatment, the treatment group was divided into pre-treatment, 3-week, 6-week, and 12-week treatment groups (Table 1). There were no significant differences in sex and age among the three subgroups (all *P* > 0.05). Approved by The Ethics Committee of Fujian Province Hospital (K2016-04-015, K2020-07-023), all patients enrolled in the study signed the informed consent form.

**Table 1:** ASDAS-CRP score in each group

	N	ASDAS-CRP	Age	Sex M: F
Control group	15	0.60 ± 0.00	17–59 (35.6 ± 13.8)	9:6
Case group	99	2.48 ± 1.27	14–71 (35.5 ± 13.6)	60:39
Inactive group	20	0.67 ± 0.12	17–54 (33.2 ± 10.6)	7:13
Active group	79	2.94 ± 0.98	14–71 (36.1 ± 14.3)	53:26
Moderate activity group	18	1.73 ± 0.26	17–71 (34.4 ± 13.2)	10:8
High disease activity group	42	2.86 ± 0.39	18–63 (36.8 ± 14.4)	30:12
Very high disease activity group	19	4.28 ± 0.63	14–70 (36.1 ± 15.6)	13:6
Pre-treatment	20	3.01 ± 0.84	17–54 (28.5 ± 9.9)	11:9
After 3W of treatment	20	1.61 ± 0.88	–	–
After 6W of treatment	20	1.11 ± 0.59	–	–
After 12W of treatment	20	0.75 ± 0.35	–	–

### Inspection method

All patients were scanned by a 1.5T MRI scanner (Magnetom Aera, SIEMENS Healthcare, Erlagnen, Germany). Sequences used included conventional transverse axial T1WI, T2WI, T2WI-fs and coronal PDWI-fs, plus coronal T1 mapping (TR 11 ms, TE 1.57 ms, flip angle 5, 27°, FOV 240 mm × 240 mm, Matrix 256 × 256, layer thickness 3.0 mm, layer number 22, interval 0.6 mm, iPAT factor 2, scanning time 2min07s), T2 mapping (TR 1200 ms, TE 13.8, 27.6, 41.4, 55.2,69.0 ms, flip angle 180°, FOV 240 mm × 240 mm, Matrix 256 × 256, layer thickness 3.5 mm, layer number 15, interval 0 mm, iPAT factor none, scanning time 5min29s), T2* mapping (TR 422 ms, TE 4.18, 11.32, 18.46, 25.60, 32.74 ms, flip angle 60°, FOV 240 mm × 240 mm, matrix 256 × 256, layer thickness 3.0 mm, layer number 15, interval 0 mm, iPAT factor 2, scanning time 3 min 46 s).

### Measurement of relaxation time

After scanning, pseudo-color images of T1 mapping, T2 mapping, and T2* mapping were automatically generated. All imaging assessments were performed on Siemens syngo MRD13 image post-processing workstation with standard software. Two senior radiologists combined pseudo-color images with conventional oblique coronal images of sacroiliac joint. In the control group and the case group, ROIs were manually delineated in the sacral and iliac subchondral bone marrow areas of bilateral sacroiliac joints. Three ROIs were selected in each quadrant, each with a size of about 25 mm^2^. The average value of ROIs of bilateral sacral sidesacroiliac joints was taken as the final measurement value of sacral side sacroiliac joints, and the average value of ROIs of bilateral iliac joints was taken as the final measurement value of iliac joints. The most obvious layer of lesion of the case group was selected and the region of interest was placed in the lesion center for measurement. The ROIs of the patients in the treatment group were placed in the corresponding area of the initial diagnosis. ROIs should be selected as close to articular cartilage as possible, but excluding articular cartilage. ROIs should also be far from blood vessels, bone cortex and other areas. In order to reduce errors, all data were measured three times. If differences of measurement data were distinct, the two radiologists would conduct the measurement at the same position on the same level and reach a final consistent result.

### Statistical analysis

SPSS (Version 25.0, Inc., Chicago, IL, USA) was applied to analyze data, and the normal test was carried out on the measurement data. Data following normal distribution was represented by means ± standard deviation (SD) and data not normally distributed was represented by median (Q1, Q3). Values of T1 mapping, T2 mapping and T2* mapping of each group were compared by rank sum test. ROC curve was used to analyze the diagnostic efficiency. Kruskal–Wallis H test was employed to analyze the variables of the three subgroups in the activity group. A paired T-test was used to compare the decreasing rate of T1 mapping value in each treatment group. The test level α = 0.05 (*P* < 0.05) was statistically significant.

## Results

### ASDAS-CRP score and routine MRI performance

ASDAS-CRP scores of the control group and the case group were evaluated by two senior physicians of the rheumatic immunology department (Table 1).

**Fig. 1:**
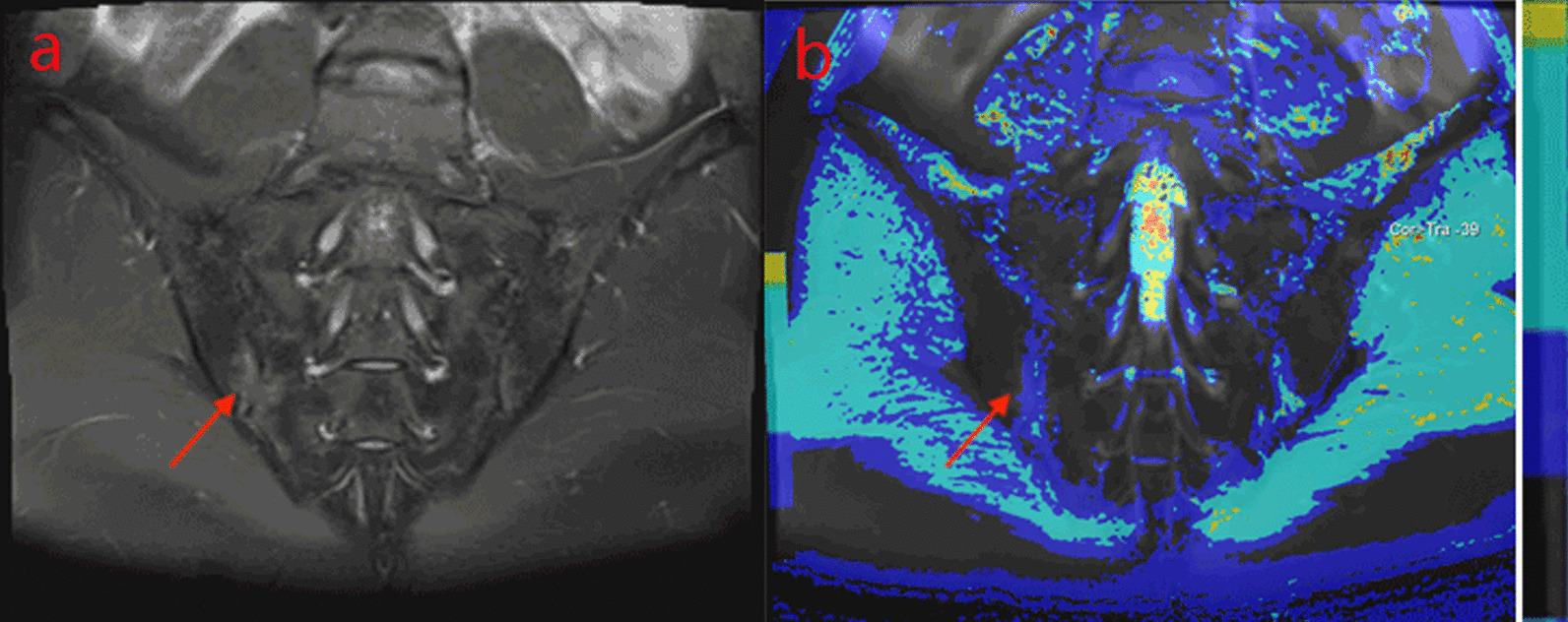
A male, 32 years old, HLA-B27: + , ASDAS-CRP:1.9, belongs to the moderate activity group. **a** The coronal plane of PDWI shows high signal intensity of bone marrow under right sacroiliac articular surface. **b** T1-mapping pseudo-color diagram demonstrates the T1-mapping values of regions of interest is 519.27 ms

**Fig. 2:**
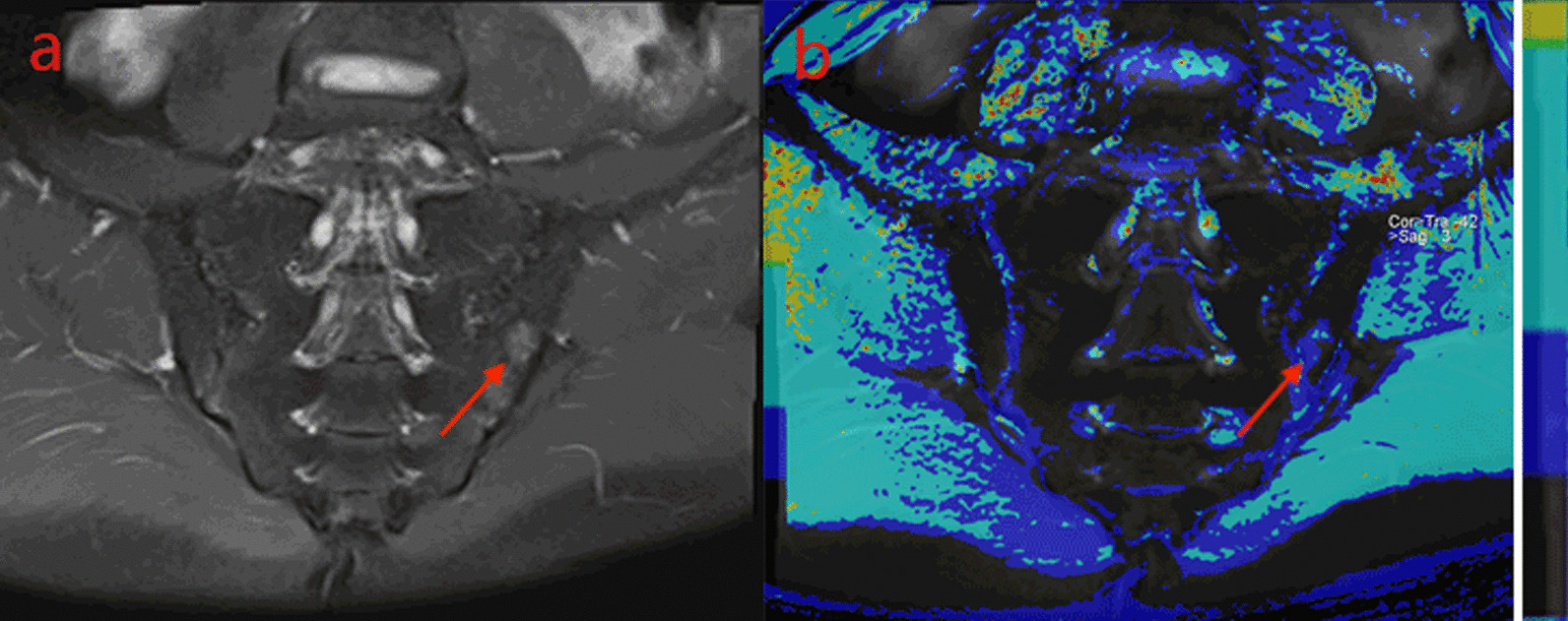
A male, 27 years old, HLA-B27: ± , ASDAS-CRP:2.5, belongs to the high disease activity group. **a** The coronal plane of PDWI shows high signal intensity of bone marrow under left sacroiliac articular surface. **b** T1-mapping pseudo-color diagram demonstrates the T1-mapping values of regions of interest is 725.77 ms

**Fig. 3:**
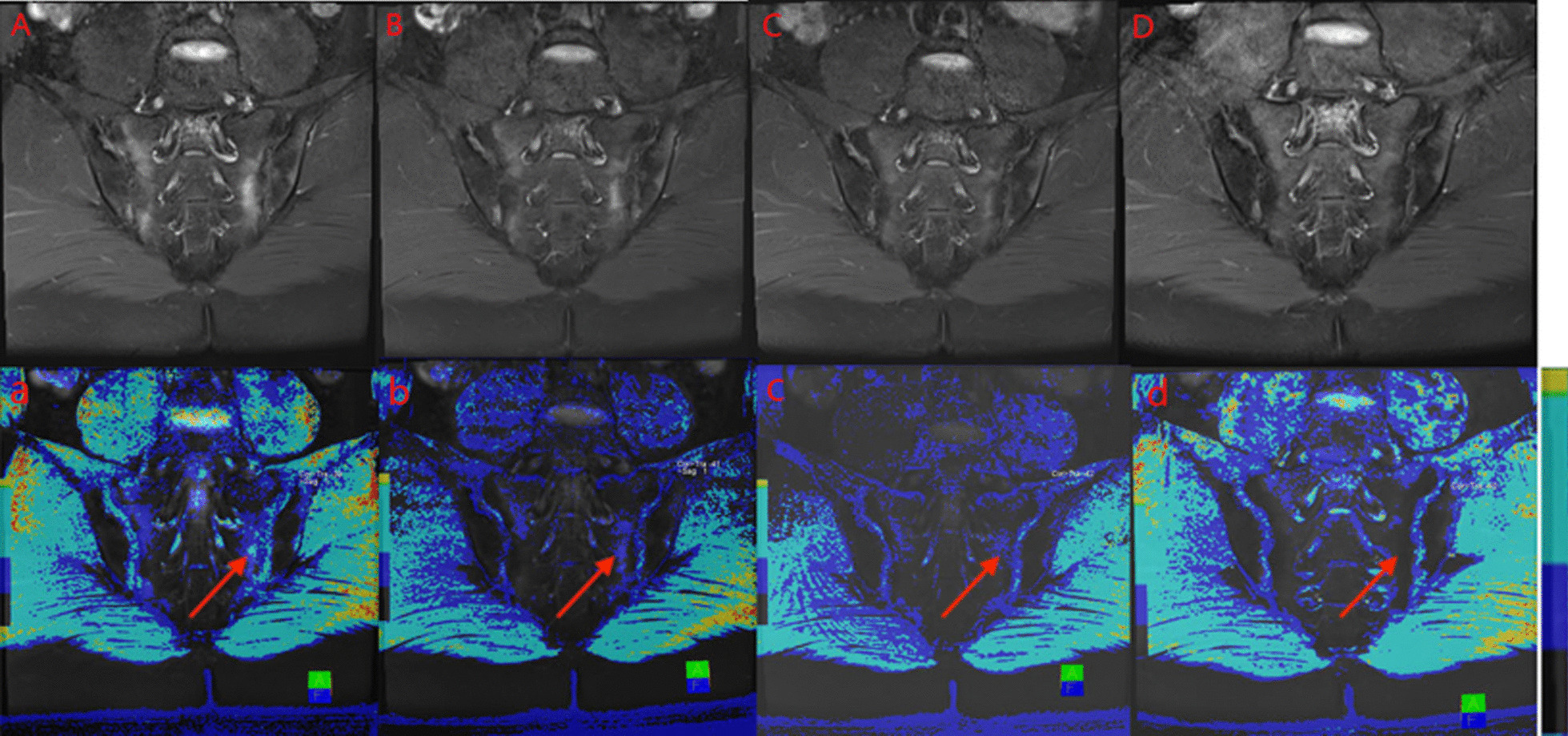
A male, 24 years old, HLA-B27: ± , ASDAS-CRP:3.8, belongs to the very high disease activity group. Depending on the different treatment cycles, these are pre-treatment (**A**,**a**), 3 weeks (**B**,**b**), 6 weeks (**C**,**c**) and 12 weeks (**D**,**d**) after treatment, respectively.** A**–**D**. Fat suppression PDWI sequences show the decreasing of sacroiliac bone marrow signal, gradually. the ASDAS-CRP score decreased with the increase of the treatment cycle (2.6, 1.6, 1.0, 0.6). **a**–**d**. T1-mapping pseudo-color diagrams also demonstrate the decreasing of the regions of interest T1-mapping value, gradually. (956.43, 780.73, 604.73, 349.27 ms).

MRI findings of the sacroiliac joint in 99 patients with SpA mainly included different degrees of BME and fat deposition and bone erosion and destruction. BME appeared hypointense on T1WI and hyperintense on PDWI-fs. As the ASDAS-CRP score increased, the degree of sacroiliac joint BME increased accordingly, and PDWI-fs showed that the signal of bone marrow under sacroiliac joint surface increased in different degrees (Figs. 1a, 2a, 3a).

### Comparison of relaxation time values of the subchondral bone marrow water content of sacral and iliac within groups

It was tested that the relaxation time values of the sacral and iliac side in the Inactive group and Active group were not normally distributed, and there was no significant difference in sacral and iliac relaxation time among the three groups (Table 2).

**Table 2:** Comparison of sacroiliac joint relaxation time

	Control group	Case group	Inactive group	Active group
T1-mapping(ms) Sacral	240.33(228.93, 274.57)	473.73(328.33, 956.43)	338.40(285.17, 404.48)	529.87(369.17, 1022.23)
T1-mapping(ms) iliac	233.67(223.93, 257.27)	480.03(351.73, 830.07)	347.55(294.34, 398.93)	598.77(370.13, 865.83)
Z	− 0.284	− 1.092	− 0.933	− 1.422
P	0.776	0.275	0.351	0.155
T2-mapping(ms) Sacral	104.63(95.13, 107.13)	107.73(93.23, 119.03)	106.73(99.44, 117.17)	108.03(90.73, 121.43)
T2-mapping(ms) iliac	101.03(93.63, 105.33)	103.23(89.37, 117.67)	111.50(97.28, 129.36)	102.23(88.67, 110.83)
Z	− 0.795	− 1.134	− 0.859	− 1.596
P	0.427	0.257	0.391	0.111
T2*-mapping(ms) Sacra	6.17(5.47, 7.13)	7.87(6.53, 9.93)	7.33(6.16, 8.18)	8.23(6.53, 10.57)
T2*-mapping(ms) iliac	6.33(5.57, 6.8)	7.63(6.53, 8.93)	7.20(6.53, 7.86)	7.83(6.67, 9.73)
Z	− 0.057	− 1.769	− 0.342	− 1.698
P	0.955	0.077	0.732	0.089

### Comparison of relaxation time values of subchondral bone marrow water content between sacroiliac joint

No matter the sacral or the iliac, values of T1 mapping and T2* mapping were significantly different among each group, but there was no significant difference in T2 mapping among the four groups (Table 3). AUC of T1 mapping was higher than that of T2* mapping among each group. T1 mapping had the best diagnostic performance, better than T2* mapping (Table 4). There was no significant difference in T2 mapping (*P* value: 0.372, 0.317, 0.430, 0.969 in sacral; 0.589, 0.161, 0.816, 0.100 in iliac).

**Table 3:** Comparison of relaxation time values of sacral and iliac of sacroiliac joint among different groups

		Sacral			Iliac		
		T1-mapping	T2-mapping	T2*-mapping	T1-mapping	T2-mapping	T2*-mapping
Control group-Case group	H	26.275	0.797	13.793	29.369	0.292	14.108
	*P*	< 0.001	0.372	< 0.001	< 0.001	0.589	< 0.001
Control group-Inactive group	H	11.560	1.000	4.772	17.640	1.961	7.024
	*P*	0.001	0.317	0.029	< 0.001	0.161	0.008
Control group- Active group	H	27.671	0.624	15.194	28.879	0.054	14.481
	*P*	< 0.001	0.430	< 0.001	< 0.001	0.816	< 0.001
Inactive group- Active group	H	16.493	0.002	5.724	13.718	2.713	3.898
	*P*	< 0.001	0.969	0.017	< 0.001	0.100	0.048

**Table 4:** Comparison of diagnostic efficacy of sacroiliac joint sacral and iliac relaxation time values

		AUC	sensitivity	specificity	*P*
Control group-case group	T1-mapping(ms) sacral	0.912	0.788	0.933	< 0.001
	T1-mapping(ms) iliac	0.935	0.869	0.933	< 0.001
	T2*-mapping(ms) sacral	0.798	0.576	0.933	< 0.001
	T2*-mapping(ms) iliac	0.802	0.556	0.933	< 0.001
Control group-inactive group	T1-mapping(ms) sacral	0.840	0.900	0.733	0.001
	T1-mapping(ms) iliac	0.920	0.900	0.867	< 0.001
	T2*-mapping(ms) sacral	0.718	0.650	0.800	0.029
	T2*-mapping(ms) iliac	0.765	0.800	0.667	0.008
Control group-active group	T1-mapping(ms) sacral	0.930	0.848	0.933	< 0.001
	T1-mapping(ms) iliac	0.939	0.899	0.933	< 0.001
	T2*-mapping(ms) sacral	0.819	0.633	0.933	< 0.001
	T2*-mapping(ms) iliac	0.811	0.582	0.933	< 0.001
Inactive group-active group	T1-mapping(ms) sacral	0.795	0.671	0.900	< 0.001
	T1-mapping(ms) iliac	0.770	0.709	0.800	< 0.001
	T2*-mapping(ms) sacral	0.674	0.595	0.750	0.017
	T2*-mapping(ms) iliac	0.643	0.430	0.950	0.048

### Comparison of relaxation time of subchondral bone marrow water content among three subgroups in the Activity group

Different relaxation time values of sacroiliac joints appeared in moderate activity group, high disease activity group, and very high disease activity group (Table 5). Values of T1 mapping (Fig. 1b, 2b, 3a), T2* mapping and T2 mapping in each subgroup of the Active group increased with the increase of disease activity, and the color deepened on the pseudo-color map. Using Kruskal–Wallis H test, the difference of T1 mapping value among each group was statistically significant. (iliac H = 11.496, *P* = 0.003; sacral H = 11.954, *P* = 0.003). After the pairwise comparison of correcting the significance level by Bonferroni method, it was found that there were significant differences in T1 mapping between moderate group and very high disease activity group (iliac *P* = 0.002, sacral *P* = 0.004) and between high disease activity group and very high disease activity group (sacral *P* = 0.018, iliac *P* = 0.029). However, there was no significant difference between moderate activity group and high disease activity group (iliac *P* = 0.494, sacral *P* = 0.878,). There was no significant difference in values of T2 mapping and T2* mapping in the subgroup of the active group (iliac *P* = 0.455, sacral *P* = 0.703), (iliac *P* = 0.191, sacral *P* = 0.457).

**Table 5:** Comparison of three MR Relaxometry technology parameters of sacroiliac joint among subgroups of the Activity group

	Moderate group	High disease activity group	Very high disease activity group
T1-mapping(ms) sacral	453.05(45.54, 05.87)	511.20(327.19, 013.62)	734.93(558.17, 221.37)
T2-mapping(ms) sacral	105.58(91.67, 17.26)	106.28(91.08, 18.50)	112.10(88.43, 37.87)
T2*-mapping(ms) sacral	8.00(6.26, 0.97)	8.15(6.53, 1.07)	8.53(6.67, 1.43)
T1-mapping(ms) iliac	365.23(330.36, 84.84)	616.15(373.00, 47.78)	848.83(528.93, 134.23)
T2-mapping(ms) iliac	103.90(88.82, 09.44)	100.20(87.77, 10.34)	105.97(90.77, 18.87)
T2*-mapping(ms) iliac	6.78(6.29, 0.72)	7.83(6.67, 0.61)	8.77(7.17, 0.83)

### Comparison of decreasing rate of T1 mapping value in the subchondral bone marrow area of the treatment group

**Fig. 4:**
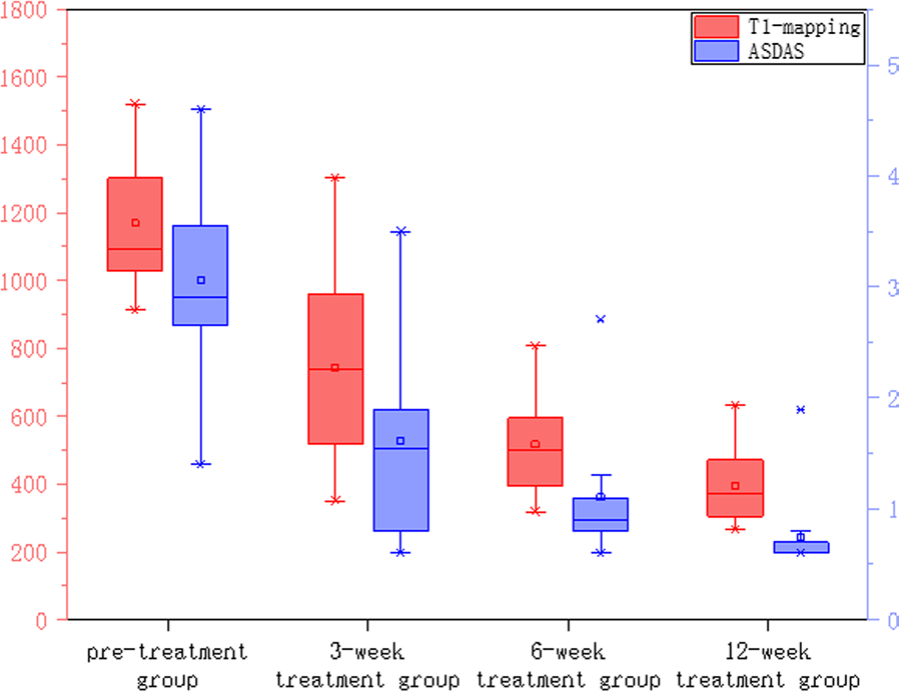
Changs in T1 mapping and ASDAS in the treatment group. The values of T1-mapping and ASDAS-CRP in the treatment group decreased synchronously pre-treatment, 3 weeks, 6 weeks and 12 weeks after treatment

In the treatment group, signal intensity of bone marrow under sacroiliac joint surface decreased in varying degrees among pre-treatment, 3-weeks, 6-weeks, and 12-weeks treatment groups. The PDWI sequence showed that the signal of subsacroiliac bone marrow decreased in different degrees (Fig. 3A–C). The value of T1 mapping showed a downward trend (Fig. 3a–c), and the value of T1 mapping decreased synchronously with the value of ASDAS-CRP (Fig. 4). Using the Spearman rank correlation analysis, the correlation coefficient between T1 mapping value and ASDAS-CRP score was 0.713, *P* < 0.001. Compared with the paired T-test, the difference of T1 mapping value in each treatment week was statistically significant, T (7.311, 5.596, 6.083), *P* < 0.001. Compared with the previous cycle, the decrease of bone marrow T1 mapping value in the 3-week, 6-week, and 12-week treatment groups were (426.60 ± 260.95, 224.47 ± 179.39, 124.92 ± 91.85), and the average decreasing rate of T1 mapping was (0.360 ± 0.203, 0.551 ± 0.129, 0.658 ± 0.098). Figure 4 the values of T1-mapping and ASDAS-CRP in the treatment group decreased synchronously pre-treatment, 3 weeks, 6 weeks and 12 weeks after treatment.

## Discussion

### Common evaluation methods and limitations of SpA active sacroiliac arthritis

ASDAS-CRP [8] score is a new SpA activity score introduced in 2009. It is the first disease activity score system that integrates subjective evaluation of patients and objective indicators of laboratory. It is currently the most accurate disease activity index related to the progress of SpA spinal imaging [9], but its calculation still depends on patients' subjective evaluation, affecting the accuracy.

Commonly used imaging methods include X-ray, CT, MRI and ultrasound. X-ray and CT can only indicate the "static" bone structural and morphological changes, and fail to show inflammation in the active stage [10]. The sensitivity and specificity of radionuclide imaging are low [11]. Ultrasound examination is easily affected by the operator's proficiency, the sonogram is easily disturbed by bones, and it is difficult to fully display the sacroiliac joint [12].

MRI STIR sequence is the most sensitive and specific method for sacroiliac joint inflammation, which directly shows the articular cartilage abnormalities, periarticular BME, and fat deposition, benefiting early detection and diagnosis of sacroiliac joint inflammation [13]. At present, STIR sequence images are only a kind of morphological imaging, the reflection of disease status still lags behind pathological changes, and cannot provide quantitative indexes.

### MR Relaxometry technology and clinical application

Early changes of sacroiliac joints in sacroiliac arthritis are mainly manifested as synovitis and inflammation of bone marrow around the joints. This is the pathological basis for the correlation between BME and disease activity state suggested by imaging.

T1 mapping imaging technology reflects the slow frequency interaction between water and extracellular matrix molecules in tissue, is sensitive to changes of proteoglycan content in tissue, and is independent from the signal intensity of reference tissue. In the past, it was used to quantify the degree of myocardial edema and fibrosis [14], because the tissue T1 value was relatively independent, not affected by the direction of collagen arrangement, and was also used for articular cartilage MR Relaxometry technology [15]. At present, there is little discussion on this at home and abroad. T1 mapping technique can detect small changes of water molecules in tissue and quantitatively evaluate the degree of subchondral BME of sacroiliac joint in SpA. In the diagnosis of BME, sensitivity, accuracy and specificity of T1 mapping are better than that of T2 mapping and T2* mapping. Furthermore in the process of early treatment, the degree of BME can be detected in real time. Therefore, it can be deduced that the accurate measurement of water content by T1 mapping quantitative technique may help to accurately evaluate the edema of early sacroiliac arthritis and changes of BME during early treatment.

T2 mapping imaging technology is related to the content of free water in the cartilage and the arrangement direction of cartilage collagen fibers, which can reflect the interaction between water molecules and collagen fibers in the cartilage, as well as the ability of proton exchange between water and hydrogen, tissue matrix structure and water content [16, 17]. It is often used to detect cartilage lesions in bone and joint lesions [17]. However, the T2 mapping value is easily affected by the external environment. Different age stages, bone marrow mineral composition, lipid deposition, and changes in the reticular structure of the bone trabeculae [18] will change the T2 mapping value [19], which explains the great fluctuation of T2 mapping value and poor diagnostic efficiency in this study.

T2* mapping imaging [5] is a multi-echo gradient volume scanning technique, which can obviously shorten the scanning time, and has the advantages of thin imaging layer, high spatial resolution, high signal-to-noise ratio, clear imaging contrast and so on. Theoretically, T2* mapping imaging is superior to T2 mapping imaging in clinical application, and has the potential to replace T2 mapping imaging as a sensitive index for early diagnosis and monitoring of articular cartilage lesions [20, 21]. In this study, T2* mapping imaging is superior to T2 mapping imaging in the diagnosis of BME, slightly lower than T1 mapping imaging (and can complement T1 mapping imaging).

This study showed that values of T1 mapping and T2* mapping in the Inactive group were higher than those in the control group, suggesting that BME may occur in sacroiliac arthritis in the Inactive stage. Some studies [22] have shown that the observation of subchondral BME of sacroiliac joint by MRI is the key to proving the active stage of AS, which is similar to the result of Lu Chuan's study on the relationship between knee joint inflammation and BME [23]. As the disease progressed, ASDAS-CRP score of the patients in the activity group increased, the corresponding sacroiliac joint BME increased, the corresponding relaxation time prolonged, and the values of T1 mapping and T2 *-mapping increased. There was no significant difference in T2 mapping among each group. Its main reasons, on the one hand, lie in the disorder of collagen fiber arrangement and the abnormal deposition of lipid caused by sacroiliac joint bone involvement due to sacroiliac arthritis. On the other hand, T2- mapping value is easily affected by diffusion-induced signal loss and magnetic susceptibility artifacts. In addition, T2 mapping imaging time is longer than T1 mapping, T2* mapping imaging, which is not conducive to clinical application. Therefore, it is suggested that T1- mapping or T2* mapping should be preferred in the quantitative diagnosis of sacroiliac joint.

### Evaluation of therapeutic effect of MR relaxometry on SpA after treatment

At present, sufficient evidence has been obtained for the clinical efficacy of TNF- α antagonists in the treatment of SpA. Clinicians have agreed on the application of biological agents as the first-line drug for patients with active SpA [2, 24, 25]. However, these biological agents for patients are expensive, increasing the economic burden of families and society, and long-term use also poses risks to potential infection and tumor. Therefore, the appropriate reduced dose treatment plan for patients after remission or LDA (low disease activity) can reduce the economic burden of the patient's family and society, reduce the side effects of the drug and maintain the curative effect. On the other hand, there are individual differences in the severity of the disease in the treatment and prognosis stages. Therefore, how to correctly evaluate changes in patients' condition and determine the efficacy of drugs has become an urgent clinical problem to be solved. Although ASDAS-CRP score is now widely used in clinical monitoring of SpA patients after treatment, it still depends on patients' subjective evaluation, making it difficult to accurately reflect the activity state of the disease. T1 mapping can reflect subtle changes of water molecules in tissues in quantitative form. In this study, it was found that after enalapril treatment, the value of T1 mapping was significantly lower than that of pre-treatment group. In the treatment group, the decreasing rate of T1 mapping value of sacroiliac joint in different treatment periods further increased with the prolongation of the treatment cycle, and the difference between groups was statistically significant(*P* < 0.05). The results showed that the degree of BME decreased in different treatment cycles, and it was further proved that TNF- α antagonist could effectively control the active state of SpA disease. In the treatment group, Spearman grade correlation analysis showed that there was a significant correlation between T1 mapping value and ASDAS-CRP score, which proved that T1 mapping value combined with ASDAS-CRP score could be used to evaluate the condition of patients after treatment with enalapril.

## Limitations of the study

(1) The pathological changes of sacroiliac arthritis are complex, including BME, fat deposition, and hyperosteogeny [26]. In this study, subchondral BME was discussed, but other factors were not discussed. (2) ASDAS-CRP score was used as the standard of grouping and curative effect evaluation after treatment, but there was no guidance of pathological gold standard. (3) Sample size of the treatment group is small, which may cause the deviation of the results, and the study lacks comparison with other monotherapy regimens, so further verification by larger samples, multicenter, randomized controlled trials is needed.

## Conclusion

MR Relaxometry technology can be used to evaluate the subchondral BME of sacroiliac joint in SpA. In quantitative diagnosis, T1 mapping technology is preferred, and T2* mapping is complementary to T1 mapping. The diagnostic efficacy of T1 mapping in subchondral BME of the sacroiliac joint of SpA was better than that of T2* mapping and T2 mapping. T1 mapping technology provides an effective quantitative index for clinical diagnosis and treatment of SpA, which is beneficial to clinical individualized treatment and timely adjustment of the treatment plan.

## Data Availability

The project is a prospective study. All cases were collected from Fujian provincial hospital. All data generated or analysed during this study are included in this published article [and its supplementary information files], presented in tables and pictures. The datasets during the current study are available from the corresponding author on reasonable request.

## Abbreviations

TNF-α: Tumor necrosis factor alpha
SpA: Ankylosing spondylarthritis
STIR: Short TI inversion recovery
ASDAS: Ankylosing spondylitis disease activity score
ROI: Regions of interest
CT: Computerized tomography
MRI: Magnetic resonance imaging
AUC: Area under curve
BME: Bone marrow edema
